# Antagonizing STAT5B dimerization with an osmium complex

**DOI:** 10.1038/srep36044

**Published:** 2016-11-17

**Authors:** Li-Juan Liu, Wanhe Wang, Tian-Shu Kang, Jia-Xin Liang, Chenfu Liu, Daniel W. J. Kwong, Vincent Kam Wai Wong, Dik-Lung Ma, Chung-Hang Leung

**Affiliations:** 1 State Key Laboratory of Quality Research in Chinese Medicine, Institute of Chinese Medical Sciences, University of Macau, Macao, China; 2 Department of Chemistry, Hong Kong Baptist University, Kowloon Tong, Hong Kong, China; 3 State Key Laboratory of Quality Research in Chinese Medicine, Macau University of Science and Technology, Taipa, Macao, China

## Abstract

Targeting STAT5 is an appealing therapeutic strategy for the treatment of hematologic malignancies and inflammation. Here, we present the novel osmium(II) complex **1** as the first metal-based inhibitor of STAT5B dimerization. Complex **1** exhibited superior inhibitory activity against STAT5B DNA binding compared to STAT5A DNA binding. Moreover, **1** repressed STAT5B transcription and blocked STAT5B dimerization via binding to the STAT5B protein, thereby inhibiting STAT5B translocation to the nucleus. Furthermore, **1** was able to selectively inhibit STAT5B phosphorylation without affecting the expression level of STAT5B.

Aberrant activation of tyrosine kinase activity has been implicated in the progression of a wide range of disorders, including cancer, inflammation, diabetes, and angiogenesis. These dysregulated kinases trigger the abnormal propagation of signal transduction to stimulate the activation of genes involved in critical biological processes via recruitment of transcription factors^1^,^2^. Among the family of transcription factors activated by tyrosine kinases, the signal transducer and activator of transcription (STAT) family has emerged as one of most important players in human cancer and inflammation^3^,^4^. These transcription factors are activated by phosphorylation on a specific tyrosine residue, and dimerize through reciprocal Src homology 2 (SH2) domain-phosphorylated tyrosine interactions^5^. After migrating to the nucleus, STATs regulate genes involved in survival, proliferation, and differentiation, through the interaction with specific response elements in DNA^6^.

The STAT5 proteins, containing the closely related members STAT5A and STAT5B that share 96% homology at the amino acid level, is frequently constitutively active in solid cancers, leukemias, and inflammation^7^,^8^,^9^. STAT5 can become tyrosine-phosphorylated at the C-terminus (Y694 for STAT5A and Y699 for STAT5B) by kinases, such as Janus kinases (JAKs), associated with transmembrane receptors, including prolactin (PRL), IL-2, GM-CSF, and growth hormone (GH) receptors^10^,^11^. PRL can modulate immune and inflammatory responses via the activation of STAT5B in T cells in the PRL-R/JAK/STAT5B/IRF-1 signaling pathway^12^, and PRL is locally expressed as a cytokine in some cancers, such as breast cancer or prostate cancer^13^,^14^, suggesting that PRL-activated STAT5B activation is a pathogenic event in tumorigenesis and inflammation^15^,^16^. Due to the role of aberrant STAT5 signaling in various human disease processes, a number of small-molecule STAT5 inhibitors towards blocking the abnormality of STAT5 through a number of direct and indirect approaches have been developed in recent years. Berg and co-workers reported nonpeptidic chromone-based compounds (STAT5i) that directly targeted the SH2 domain of STAT5 and selectively disrupted the STAT5 SH2 domain-phosphorylated tyrosine interaction over that of STAT1/3, leading an inhibition of STAT5/DNA binding in K562 nuclear extracts^17^. Recently, a potent molecule derived from the natural product-dihydrocapsaicin inhibited the SH2 domain of STAT5B, with 35-fold selectivity over STAT5A^18^. However, to date, no direct STAT5B inhibitors have entered into clinical trials. Alternatively, indirect inhibitors of STAT5 that target upstream signaling proteins such as Bcr-Abl, Jak2 and FLT-3 inhibitors have also been reported^19^.

The rapid development of metal complexes in medicine has attracted worldwide attention. Many of the metal complexes have been utilized for diagnostic and treatment purposes^20^,^21^,^22^,^23^,^24^,^25^,^26^,^27^,^28^,^29^,^30^,^31^,^32^. Metal complexes are endowed with unique characteristics, including facile synthesis, tunable properties, specific reactivity towards biomolecular targets, and redox activity^33^,^34^. Osmium complexes, with highly toxic activity, are less common in the medical application. However, several osmium complexes, structurally close analogues of the antimetastatic complex NAMI-A^35^, have been reported to exert reasonable antiproliferative activity *in vitro*^36^. In 2008, Meggers and co-workers revealed the promising protein kinases GSK-3β and Pim-1 inhibition and anticancer effects towards melanoma cells of the osmium half-sandwich complex^37^. This work highlighted the concept of designing unreactive bioactive metal complexes. Additionally, organometallic osmium(II) complexes, such as FY26, [Os(η^6^-p-cym)(p-NMe_2_-Azpy)I]PF_6_, increased the levels of reactive oxygen species (ROS) produced in cancer cells, allowing FY26 to exert selective toxicity toward cancer cells over normal cells^38^. A novel osmium-based organometallic compound, [Os_3_(CO)10(μ-H)(μ-S)C_9_H_6_N], has found to target the mitochondria and triggers ROS-dependent apoptosis *in virto* and *in vivo*, as well as inhibit tumor growth in colon carcinoma^39^. In the context of STAT inhibition, certain metal-based complexes have shown anticancer activity by means of STAT inhibition^40^,^41^. For example, Priebe *et al.* described a family of gold-containing metal complexes which abolished STAT3 phosphorylation^42^, while our group have discovered a rhodium-based metal complex that directly and effectively targets the STAT3 SH2 domain^43^. However, to our knowledge, no metal-based inhibitors have been reported to inhibit STAT5 function.

Protein-protein interfaces are generally regarded to be relatively amorphous, lacking the specific contacts that are often found in enzyme or receptor binding sites. Consequently, we envisaged that an osmium(II) complex, carrying relatively hydrophobic ligands in a well-defined octahedral geometry, might possess a suitable shape to interact at the protein-protein interaction region of STAT5B. We describe herein the identification and characterization of a novel osmium(II) metal-containing complex, which potently binds to STAT5B and inhibits the STAT5B signaling pathway. These findings provide a new framework for STAT5B inhibition by metal complexes as a potential strategy for anti-cancer or anti-inflammatory therapy.

## Results and Discussion

### Chemical syntheses

**Figure 1:**
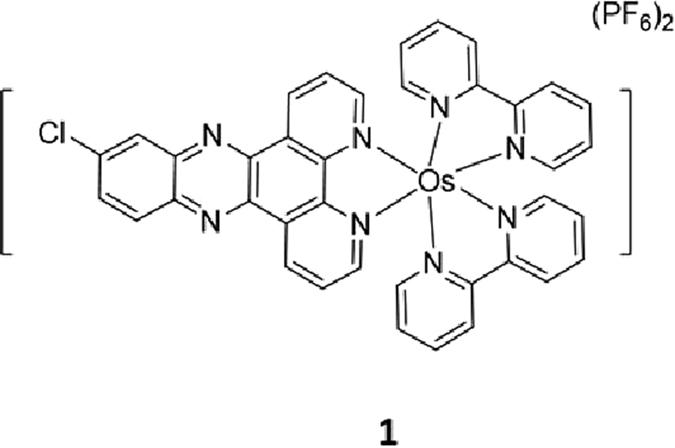
Chemical structure of osmium(II) complex 1.

**Figure 2:**
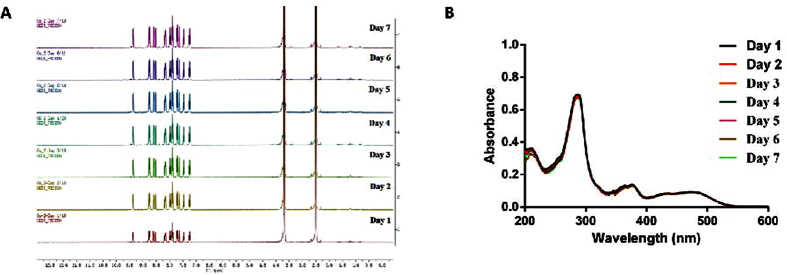
The stability of complex 1. (**A**) The ^1^H NMR spectra of **1** at a concentration of 5 mM in 90% [*d*_6_] DMSO/10% D_2_O at 298 K over 7 days. (**B**) UV/Vis absorption of **1** at a concentration of 10 μM in 80% acetonitrile/20% 20 mM Tris-HCl buffer (containing 20 mM NaCl, pH 7.5) at 298 K over 7 days.

In order to discover novel metal-containing complex targeting STAT5B activity, osmium(II) complex **1** (Fig. 1) with a general structure [Os(bpy)_2_(N^N)](PF_6_)_2_ (where bpy = 2,2′-bipyridine) was designed and synthesized. Complex **1** bear dipyrido[3,2-a:2′,3′-c]phenazine (dppz) N^N ligands substituted with one chloro groups. The osmium complex was prepared in two steps using a similar method to that reported in the literature^44^. Os(bpy)_2_Cl_2_ was obtained by the reaction of (NH_4_)_2_[OsCl_6_] with bpy. Os(bpy)_2_Cl_2_ was then reacted with the different N^N ligands, followed by the addition of NH_4_PF_6_ to precipitate the crude product. Purification by column chromatography on basic alumina using acetonitrile as the eluent afforded the desired products. The complex was characterized by ^1^H-NMR, ^13^C-NMR, high resolution mass spectrometry (HRMS) and elemental analysis. The complex was stable at a concentration of 10 μM in 80% acetonitrile/20% 20 mM Tris-HCl buffer (containing 20 mM NaCl, pH 7.5) at 298 K over 7 days (Fig. 2).

### Identification of 1 as a potent STAT5B inhibitor

**Figure 3:**
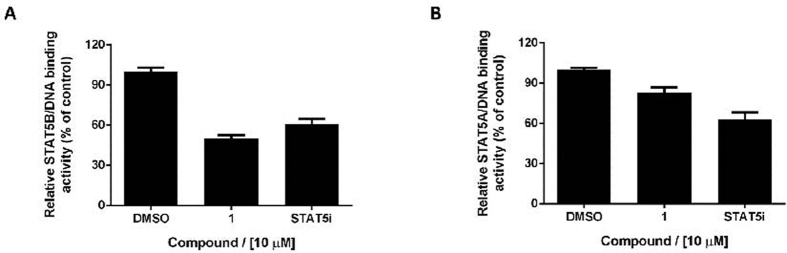
Complex 1 selectively inhibits STAT5B-DNA interaction over STAT5A-DNA interaction. (**A**) Effect of osmium(II) complex **1** on STAT5B-DNA interaction as measured by an ELISA. Complex **1** (10 μM) was treated with nuclear extract containing active STAT5B, and STAT5B binding was detected using anti-STAT5B primary antibody and horseradish peroxidase-conjugated secondary antibody. (**B**) Effect of osmium(II) complex **1** and STAT5i on STAT5A-DNA interaction as measured by an ELISA. Nuclear extract containing active STAT5A were treated with compounds (10 μM), and STAT5A binding was detected using anti-STAT5A primary antibody and horseradish peroxidase-conjugated secondary antibody. Results are representative of three independent experiments.

To study the potential effect of the Os(II) complex on STAT5B activity, we performed a STAT5-DNA binding assay. Nuclear extract containing active STAT5B protein was pre-treated with **1** at 10 μM for 15 min. The mixture was then incubated with a double-stranded oligonucleotide containing the STAT5B consensus site, and the binding of STAT5B to the DNA was then detected by an ELISA. As shown in Fig. 3A, **1** reduced STAT5B DNA binding by 50% at 10 μM. We also evaluated the effect of **1** on STAT5A DNA binding activity. Interestingly, **1** inhibited STAT5A-DNA binding activity by less than 20% under comparable conditions (Fig. 3B). This result suggests that **1** selectively blocks the interaction between STAT5B and DNA over that of STAT5A and DNA.

### 1 antagonizes STAT5B-driven transcriptional activity in living cells

**Figure 4:**
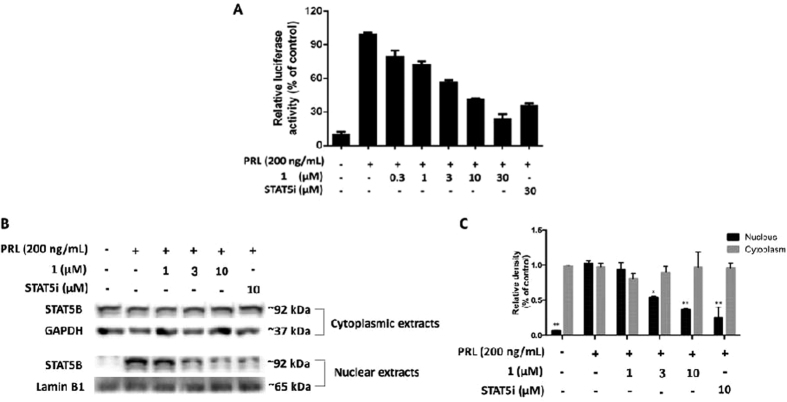
Complex 1 inhibits STAT5B-driven luciferase activity and STAT5B translocation in Caco-2 cells. (**A**) Effect of osmium(II) complex **1** on STAT5B-driven luciferase activity. Complex **1** inhibits STAT5B-driven transcription activity in PRL-induced Caco-2 cells as determined using a dual luciferase reporter assay. (**B**) Effect of osmium(II) complex **1** on STAT5B translocation. Complex **1** blocks translocation of active STAT5B from the cytoplasm to the nucleus in PRL-induced Caco-2 cells. (**C**) Densitometry analysis for the Western blotting shows inhibition of complex **1** on the translocation of active STAT5B. The data were normalized to the STAT5B expression of control group stimulated with PRL (200 ng/mL) and are expressed as the means ± SEM of three individual experiments. The data were analyzed using Image Lab. Error bars represent the standard deviation of triplicate results. *P < 0.05, **P < 0.001.

Encouraged by the ability of **1** to block STAT5B DNA-binding, we next investigated whether **1** could antagonize STAT5B-driven transcriptional activity in living cells. Using a dual firefly/*Renilla* luciferase system, we studied the levels of STAT5B-driven transcriptional activity after exposure of transfected Caco-2 cells to **1**. Caco-2 cells were incubated with **1** or the positive control compound, a nicotinoyl hydrazone STAT5B inhibitor (STAT5i)^17^ for 6 h and stimulated with PRL for a further 1 h. The results revealed that **1** attenuated STAT5B-directed transcription in a dose-dependent fashion, with an IC_50_ value of *ca.* 15 μM (Fig. 4A). Notably, the potency of **1** at inhibiting STAT5B-driven transcription at 30 μM was superior to that of STAT5i under the same conditions. These results indicate that **1** could inhibit STAT5B-directed transcription in living cells, and is consistent with its effects on the STAT5B-DNA interaction as described above. Additionally, the translocation of STAT5B in Caco-2 cells was impaired by treatment with **1**. When cells were exposed to increasing concentrations of **1**, STAT5B content in the nuclear extract was reduced in a dose-dependent manner, while STAT5B content in the cytoplasmic extract was enhanced (Fig. 4B,C). Taken together, these findings suggest that **1** is able to block the translocation of the active form of STAT5B from the cytoplasm to the nucleus, thus preventing it from binding to its corresponding response element in DNA and resulting in the repression of STAT5B-driven transcriptional activity.

### 1 disrupts the STAT5B dimerization and stabilizes STAT5B *in cellulo*

**Figure 5:**
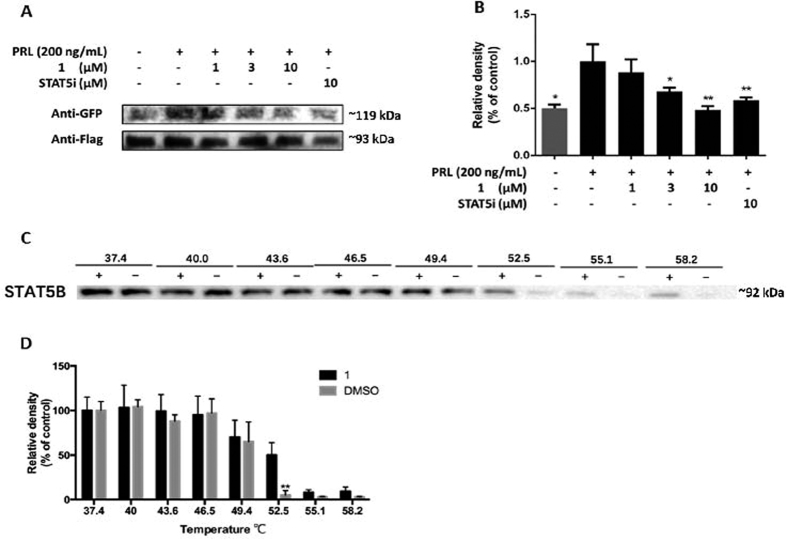
Complex 1 inhibits STAT5B dimerization and stabilizes STAT5B in Caco-2 cells. (**A**) Effect of osmium(II) complex **1** on STAT5B dimerization. Complex **1** dose-dependently inhibits the dimerization of STAT5B in Caco-2 cells co-transfected with pSTAT5B-GFP and pSTAT5B-Flag. (**B**) Densitometry analysis for the western blotting shows inhibition of complex **1** on STAT5B dimerization. The band of STAT5B-GFP was first normalized to the corresponding STAT5B-Flag in each group, and then compared to the STAT5B-GFP that from the group only treated with PRL. The data were analyzed using Image Lab. Error bars represent the standard deviation of triplicate results. (**C**) Stabilization of STAT5B by osmium(II) complex **1**. (**D**) The band intensity of STAT5B with different temperature. The data were normalized to the STAT5B expression of control group at 37.4 °C and are expressed as the means ± SEM of three individual experiments. The data were analyzed using Image Lab. Error bars represent the standard deviation of triplicate results. *P < 0.05, **P < 0.001.

We next evaluated whether **1** could directly inhibit STAT5B dimerization *in cellulo* by using a pull-down assay. Caco-2 cells overexpressing STAT5B-Flag and STAT5B-GFP were pre-treated with the indicated concentrations of **1** and positive control compound for 6 h. After 1 h simulation by PRL, proteins lysates were purified by anti-Flag magnetic beads and subsequently analyzed by immunoblotting with anti-Flag and anti-GFP antibodies. The results showed that STAT5B-GFP and STAT5B-Flag proteins were pulled down together in the absence of **1** (Fig. 5A,B). However, the level of STAT5B-GFP in the anti-Flag immunoprecipitates decreased as the concentration of **1** was increased. This result indicates that **1** disrupted STAT5B dimerization in a dose-dependent manner *in cellulo*. We hypothesized that the inhibition of intracellular STAT5B dimerization by **1** may be attributed to the direct binding of **1** to STAT5B. Therefore, we also performed a cellular thermal shift assay (CETSA)^45^ to investigate STAT5B engagement by **1** in cell lysates. Cell lysates were incubated with **1** for 30 min, then aliquots were heated at temperatures ranging from 37.4–58.2 °C followed by centrifugation, and STAT5B levels in the soluble fraction were quantified by immunoblotting. An obvious shift of the melting temperature of STAT5B protein was detected (Fig. 5C,D), indicating the thermal stabilization of STAT5B proteins in cell lysate by **1**.

### 1 selectively inhibits STAT5B phosphorylation without affecting total STAT5B expression

**Figure 6:**
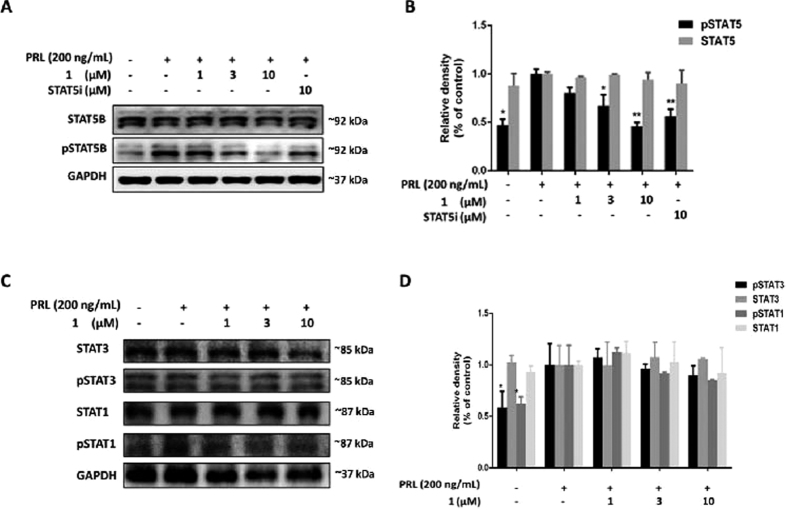
Complex 1 selectively inhibits STAT5B phosphorylation over STAT1/3 phosphorylation in Caco-2 cells. (**A**) Effect of osmium(II) complex **1** on STAT5B phosphorylation. Complex **1** inhibits PRL-induced STAT5B phosphorylation but not PRL-induced total STAT5B. (**B**) Densitometry analysis for the Western blotting shows inhibition of complex **1** on STAT5B phosphorylation. The data were normalized to the GAPDH expression and analyzed using Image Lab. (**C**) Effect of osmium(II) complex **1** on STAT1/3 phosphorylation. Complex **1** slightly inhibits PRL-induced STAT1/3 phosphorylation but not PRL-induced total STAT1/3. (**D**) Densitometry analysis shows effect of complex **1** on STAT1/3 phosphorylation and total STAT1/3. The data were normalized to the GAPDH expression and analyzed using Image Lab. Error bars represent the standard deviation of triplicate results. *P < 0.05, **P < 0.001.

**Figure 7:**
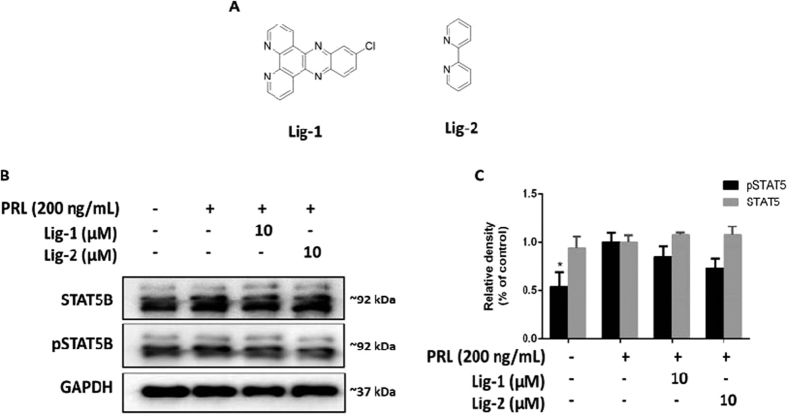
Effect of ligands of osmium(II) complex 1 on STAT5B phosphorylation. (**A**) Chemical structures of Lig-**1** and Lig-**2**. (**B**) Lig-**1** and Lig-**2** (10 μM) inhibits PRL-induced STAT5B phosphorylation but not PRL-induced total STAT5B. (**C**) Densitometry analysis shows effects of ligands **1** and **2** on STAT5B phosphorylation and total STAT5B. The data were normalized to the GAPDH expression and analyzed using Image Lab. Error bars represent the standard deviation of triplicate results. *P < 0.05, **P < 0.001.

**Figure 8:**
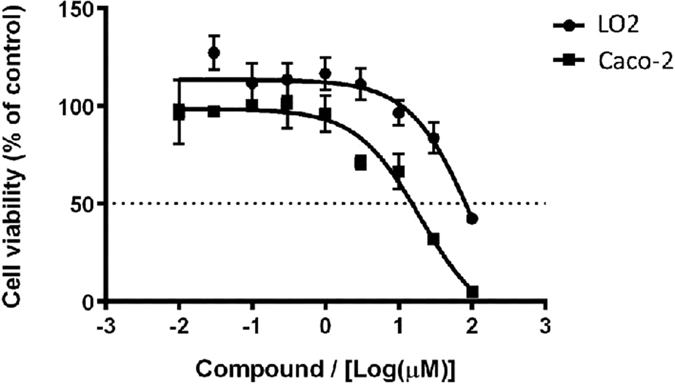
Dose-response effect of complex **1** on cell viability of Caco-2 cells and LO2 cells as determined by a MTT assay. The Caco-2 cells and LO2 cells were treated different concentrations of complex **1** for 72 h. IC_50_ values of complex **1** on Caco-2 cells and LO2 cells: 19.5 ± 1.4 μM and ~100 μM. Error bars represent the standard deviation of triplicate results.

Next, we investigated the effect of **1** on STAT5B tyrosine phosphorylation in cells. Caco-2 cells were treated with the indicated concentrations of **1** for 6 h and were further stimulated with PRL for 1 h. As expected, PRL increased STAT5B phosphorylation in Caco-2 cells. However, a dose-dependent decline in the phosphorylation level of STAT5B was observed when the cells were pre-treated with **1** or the reported STAT5 inhibitor (Fig. 6A,B). Additionally, **1** had no significant effect on total STAT5B expression. We also compared the selectivity of **1** for STAT5B over other STAT family proteins, such as STAT1 and STAT3. **1** only showed a slight inhibitory ability against STAT1/3 phosphorylation, and no obvious impact of **1** on STAT1/3 total content expression could be observed (Fig. 6C,D). To further investigate the whether the biological activity of **1** required the assembly of ligands into a three-dimensional complex, we analyzed the effect of the isolated ligands of **1** on STAT5B phosphorylation (Fig. 7). STAT5B phosphorylation was only slightly reduced by the isolated ligands, while no significant inhibitory effect on total STAT5B content could be detected. These findings indicate that the biological potency of **1** requires the arrangement of ligands into a three-dimensional structure that can interact effectively with the STAT5B protein. Finally, the complex was moderately toxic towards Caco-2 cells, with an IC_50_ value of 19.5 ± 1.4 μM, but was less toxic against LO2 cells (IC_50_ value ~100 μM) (Fig. 8). This suggests that complex **1** could be a potential STAT5B inhibitor for anticancer therapy.

## Conclusion

In this study, we have presented the novel osmium(II) complex **1** as the first metal-based inhibitor of STAT5B dimerization. Compared to STAT5i, the osmium(II) complex **1** showed similar *in vitro* STAT5B DNA-binding inhibition, but superior potency against STAT5B phosphorylation, STAT5 dimerization and transcription inhibition in cellulo. A cellular thermal shift assay revealed that **1** engaged STAT5B in cellular lysates, suggesting that **1** inhibits STAT5B dimerization through direct binding to the STAT5B protein. Furthermore, **1** was able to inhibit the phosphorylation of STAT5B without affecting STAT5B expression, block the nuclear translocation of STAT5B, and attenuate STAT5B-driven transcription activity. We envisage that the osmium(II) complex **1** may be utilized as a useful scaffold for the development of more efficacious STAT5B inhibitors.

## Materials and Methods

### General experiment

Mass spectrometry was performed at the Mass Spectroscopy Unit at the Department of Chemistry, Hong Kong Baptist University, Hong Kong (China). Deuterated solvents for NMR purposes were obtained from Armar and used as received. ^1^H and ^13^C NMR were recorded on a Bruker Avance 400 spectrometer operating at 400 MHz (^1^H) and 100 MHz (^13^C). ^1^H and ^13^C chemical shifts were referenced internally to solvent shift (Acetonitrile-d3: ^1^H, 1.94, ^13^C, 118.7). Chemical shifts (δ) are quoted in ppm, the downfield direction being defined as positive. Uncertainties in chemical shifts are typically ±0.01 ppm for ^1^H and ±0.05 for ^13^C. Coupling constants are typically ±0.1 Hz for ^1^H-^1^H and ±0.5 Hz for ^1^H-^13^C couplings. The following abbreviations are used for convenience in reporting the multiplicity of NMR resonances: s, singlet; d, doublet; t, triplet; q, quartet; m, multiplet; br, broad. All NMR data was acquired and processed using standard Bruker software (Topspin).

### Synthesis of osmium complex

#### Preparation of Os(bpy)_2_Cl_2_

A DMF solution of (NH_4_)_2_[OsCl_6_] (1 mmol, 1 eq) and 2,2′-bipyridine (2.1 mmol, 2.1 eq) was refluxed under an N_2_ atmosphere for 5 h. After cooling to room temperature, the solution was added to an aqueous solution containing Na_2_S_2_O_4_ (6.8 g) and then kept in a refrigerator overnight. The dark precipitate was collected by vacuum filtration, washed with water and diethyl ether, and directly used in the following reactions without further purification.

#### Preparation of [Os(bpy)_2_(N^N)](PF_6_)_2_

Os(bpy)_2_Cl_2_ (0.10 mmol, 1 eq) and the corresponding N^N (0.21 mmol, 0.21 eq) ligand were suspended in ethanol−H_2_O (14 mL, 5:2 v/v) in flask equipped with a magnetic stir bar. The mixture was heated to reflux and stirred for 5 h under a N_2_ atmosphere. Following the reaction, the mixture was cooled to room temperature, excess amount of NH_4_PF_6_ was added to precipitate the product as the hexafluorophosphate salt. The precipitate was washed with water and diethyl ether to give the crude product, which was purified by column chromatography on basic alumina using acetonitrile as the eluent.

**1** (Yield: 28%) ^1^H NMR (400 MHz, Acetonitrile-*d*_3_) δ 9.45–9.42 (m, 2H), 8.85 (d, *J* = 8.4 Hz, 2H), 8.81 (d, *J* = 8.0 Hz, 2H), 8.50–8.46 (m, 3H), 8.42 (d, *J* = 2.0 Hz, 1H), 8.13–8.05 (m, 5H), 8.03–7.96 (m, 6H), 7.58–7.55 (m, 2H), 7.36–7.31 (m, 2H); ^13^C NMR (100 MHz, Acetonitrile-*d*_3_) 160.2, 160.1, 154.4, 154.3, 154.2, 154.1, 152.4, 152.1, 143.5, 142.1, 141.9, 141.4, 138.7, 138.6, 138.5, 134.1, 134.0, 133.9, 132.3, 131.8, 131.7, 129.3, 129.2, 129.1, 129.0, 122.6, 125.6; MALDI-TOF-HRMS: Calcd. for C_38_H_25_N_8_ClOsPF_6_[M–PF_6_]^+^: 965.1147 Found: 965.0513; Anal.: (C_38_H_25_N_8_ClOsP_2_F_12_ + H_2_O) C, H, N: calcd. 40.49, 2.41, 9.94; found 40.40, 2.65, 9.86.

### Stability Analysis

Complex **1** was stored in [d_6_] DMSO/D_2_O (v/v = 9:1) at 298K for seven days, and was determined by ^1^H NMR spectroscopy. ^1^H NMR experiments were carried out on a 400 MHz (1H) Bruker instrument. Additionally, complex **1** was also stored in acetonitrile/Tris-HCl buffer (v/v = 8:2, 10 μM) 298K for seven days. Absorption spectra were recorded on Cary UV-100 Spectrophotometer.

### Materials

TransAM^®^ STAT5 transcription factor assay kit was purchased from Active Motif (Carlsbad, CA). TurboFect Transfection Reagent was purchased from Thermo Scientific (Hudson, NH, USA). Dual-luciferase reporter assay system, pGL-STAT5 and pRL-TK vector were obtained from Promega Corporation (Promega, Madison, WI, USA). The plasmid encoding with STAT5B gene (pCMV6-AC-GFP-STAT5B) and anti-GFP antibody were purchased from OriGene Technologies (Rockville, MD, USA). Prolactin, ANTI-FLAG^®^ M2 Magnetic Beads, Monoclonal ANTI-FLAG^®^ M2 antibody and MTT 3-(4,5-dimethylthiazol-2-yl)-2,5-diphenyltetrazolium bromide were purchased from Sigma-Aldrich (St. Louis, MO, USA).

### Cell lines

The human intestinal Caco-2 cells and the human immortal liver LO2 cells were cultivated and maintained in DMEM supplemented with 10% fetal bovine serum and 1% penicillin-streptomycin-glutamine. The Caco-2 cells were seeded in 6-well plates at a density of 1 × 10^6^ mL^–1^ in complete medium for the further experiments.

### Plasmid construction

To construct plasmids encoding the flag-fusion STAT5B proteins, the cDNAs of STAT5B were fused with flag by using an adapter ligation-mediated PCR method with primer set encoding with flag protein 5′-CGCGTGATTACAAGGATGACGACGATAAGTAAGTTT-3′ and the reverse primer is 5′-AAACTTACTTATCGTCGTCATCCTTGTAATCA-3′.

### STAT5A/B DNA-binding ELISA

The STAT5A/B DNA-binding assay was performed using the TransAM^®^ Transcription Factor ELISA (Active Motif, Carlsbad, CA) according to the manufacturer’s instructions. Briefly, nuclear extract (2 μg) containing activate STAT5A/B was added with complex (20 μL) and complete binding buffer (30 μL) to microtitre wells coated with the STAT5A/B consensus sequence. The mixture was incubated at room temperature for 1 h. The wells were washed three times with 1X wash buffer, and incubated with STAT5A/B antibodies for 1 h. The wells were washed as before and incubated with horseradish peroxide-conjugated secondary antibody for 1 h at room temperature. The wells were washed as before, incubated with 100 μL of developing solution, quenched with 100 μL stop solution, and the absorbance was measured at 450 nm.

### Pull-down assay

Caco-2 cells were co-transfected with pCMV6-AC-GFP-STAT5B and pCMV6-AC-STAT5B-flag vectors for 12 h using TurboFect Transfection Reagent. Cells were then grown in complete medium for a further 18 h. The cells were pre-treated with **1** (10 μM) or vehicle control. 6 h after pre-treatment, prolactin (200 ng/mL) was added into the well and the cells were incubated for further 30 min. The cell lysates were collected and protein concentration was determined using bicinchoninic acid (BCA) protein assay kit (Pierce, Rockford, IL, USA). 30 μg of each protein sample were pulled down with anti-Flag magnetic beads according to the instruction provided by manufacturer. The beads were equilibrated by TBS (50 mM Tris HCl, 150 mM NaCl, pH 7.4) buffer twice. Protein sample were then added to the magnetic beads and incubated for 1 h at room temperature. After 1 h, the beads were collected and the supernatant was removed. In order to remove non-specifically bound proteins, the protein-binding beads were then washed with TBS buffer twice. The protein-binding beads were then collected and Flag protein were eluted with SDS-PAGE sample buffer. Protein samples were separated by the sodium dodecyl sulfate-polyacrylamide gel electrophoresis (SDS-PAGE), and analyzed by immunoblotting by probing with anti-FLAG (1:1,000 dilution) or anti-GFP antibodies (1:1,000 dilution).

### Immunoblotting

Caco-2 cells were pre-treated with different concentrations of **1** in low FBS medium for 6 h and subsequently induced by prolactin (200 ng/mL) for another 30 min. 30 μg of each protein sample were subjected to SDS-PAGE and electro-transferred to a Immun-Blot^®^ PVDF membrane (Bio-Rad, CA, USA). Blocking with 5% non-fat milk in TBST buffer (TBST containing TBS buffer and 0.1% Tween 20) for 1 h at room temperature, the membranes were incubated with corresponding primary antibodies (anti-phospho-STAT5, anti-STAT5, anti-GAPDH, anti-phospho-STAT1, anti-STAT1, anti-phospho-STAT3 and anti-STAT3, Cell Signaling Technology, 1:1,000 dilution), washed with TBST buffer for 30 min and subsequently incubated with secondary antibodies. The signals were visualized by Image Lab. For the translocation assay, the cytoplasmic extracts and nuclear extracts were prepared by the NE-PER nuclear and cytoplasmic extraction kit following the instruction from the manufacturers. The drug-treated cells were harvested and lysed in ice-cold CER I for 10 min. The lysates were treated with CER II for 1 min and the cytoplasmic extracts in the supernatant were collected after centrifugation. The nuclear extracts were will be collected in the supernatant after the cell pellet was incubated with ice-cold NER for 40 min and subsequently centrifuged. The cytoplasmic extracts and nuclear extracts were then subjected to SDS-PAGE and analyzed by probing with STAT5B antibody and visualizing by Image Lab.

### Cellular thermal shift assay (CETSA)

Cellular thermal shift assay was performed to monitor the target engagement of **1** in Caco-2 cells. Briefly, a total 2 × 10^6^ cell numbers of Caco-2 cell lysates were collected, diluted in PBS and separated in same aliquots. Each aliquot was treated with **1** (10 μM) or DMSO. 30 min after incubation at room temperature, the complex-treated lysates were divided into 50 μL in each of PCR tubes and heated individually at different temperatures (Veriti thermal cycler, Applied Biosystems/Life Technologies). The heated lysates were centrifuged and the supernatants were analyzed SDS-PAGE followed by immunoblotting analysis by probing with anti-STAT5B antibody (Active Motif, 1:1,000 dilution).

### Luciferase reporter assay

Caco-2 cells were co-transfected with a ratio of 10:1 pGL-STAT5 and pRL-TK as a transfection efficiency control in serum-free DMEM medium using TurboFect Transfection Reagent. The transfected cells were then seeded in a 24-well plate and treated with the different concentrations of **1** in low FBS medium for 6 h and simulated with prolactin (200 ng/mL) for next 30 min. Cell lysates were then collected by 100 μL Passive Lysis Buffer (PLB). 50 μL of the cell lysates were transferred to a 96-well white plate and mixed with 50 μL of luciferase reporter reagent (LAR). Firefly luciferase activity of each well was measured in a luminometer over 10-second measurement periods using Molecular Devices SpectraMax^®^ M5 Microplate Reader. After firefly luciferase activity measurement, same volume of Stop & Glo^®^ Reagent was added to the well to quantitate *Renilla* luciferase activity at the same reading condition.

### Cell viability assay

Caco-2 cells and LO2 cells were seeded at 10,000 cells per well in 96-well plates and incubated overnight at 37 °C. Serial dilutions of complex **1** were added to each well and the plates were incubated at 37 °C in a humidified CO_2_ incubator for further 72 h. 100 μL of MTT (3-(4,5-dimethylthiazol-2-yl)-2,5-tetrazolium bromide) reagent (1 mg/mL) was added to each well. After 4 h incubation, the medium was replaced with 100 μL of DMSO and the plates were incubated at 25 °C for 10 min with shaking. Color intensity was measured at 570 nm using a microplate reader. The IC_50_ values of complex **1** were determined by the dose-dependence of the surviving cells after exposure 72 h.

### Statistics analysis

One-way analysis of variance (ANOVA) followed by the Dunnett’s method for multiple comparisons by using GraphPad Prism 6.0 was used to analyse the data. Statistically significant result was defined as *P* < 0.05.

## Additional Information

**How to cite this article**: Liu, L.-J. *et al.* Antagonizing STAT5B dimerization with an osmium complex. *Sci. Rep.* **6**, 36044; doi: 10.1038/srep36044 (2016).

**Publisher’s note**: Springer Nature remains neutral with regard to jurisdictional claims in published maps and institutional affiliations.

## References

[b1] Gschwind A., Fischer O. M. & Ullrich A. The discovery of receptor tyrosine kinases: targets for cancer therapy. Nat Rev Cancer 4, 361–370 (2004). 15122207 10.1038/nrc1360

[b2] Gross S., Rahal R., Stransky N., Lengauer C. & Hoeflich K. P. Targeting cancer with kinase inhibitors. J Clin Invest 125, 1780–1789 (2015). 25932675 10.1172/JCI76094 PMC4463189

[b3] Turkson J. STAT proteins as novel targets for cancer drug discovery. Expert Opin Ther Targets 8, 409–422 (2004). 15469392 10.1517/14728222.8.5.409

[b4] Buettner R., Mora L. B. & Jove R. Activated STAT signaling in human tumors provides novel molecular targets for therapeutic intervention. Clin Cancer Res 8, 945–954 (2002). 11948098

[b5] Bae J. H. *et al.* The Selectivity of Receptor Tyrosine Kinase Signaling Is Controlled by a Secondary SH2 Domain Binding Site. Cell 138, 514–524 (2009). 19665973 10.1016/j.cell.2009.05.028 PMC4764080

[b6] Ihle J. N. The Stat family in cytokine signaling. Curr Opin Chem Biol 13, 211–217 (2001). 10.1016/s0955-0674(00)00199-x 11248555

[b7] Vainchenker W. & Constantinescu S. N. JAK/STAT signaling in hematological malignancies. Oncogene 32, 2601–2613 (2013). 22869151 10.1038/onc.2012.347

[b8] Schwaller J. *et al.* Stat5 is essential for the myelo- and lymphoproliferative disease induced by TEL/JAK2. Mol Cell 6, 693–704 (2000). 11030348 10.1016/s1097-2765(00)00067-8

[b9] Bromberg J. Stat proteins and oncogenesis. J Clin Invest 109, 1139–1142 (2002). 11994401 10.1172/JCI15617 PMC150969

[b10] Katsantoni E. Protein Complexes and Target Genes Identification by *in Vivo* Biotinylation: The STAT5 Paradigm. Science Signaling 5 (2012). 10.1126/scisignal.2003622 23112345

[b11] Grimley P. M., Dong F. & Rui H. Stat5a and Stat5b: fraternal twins of signal transduction and transcriptional activation. Cytokine Growth Factor Rev 10, 131–157 (1999). 10743504 10.1016/s1359-6101(99)00011-8

[b12] Yu-Lee L. Y. Prolactin modulation of immune and inflammatory responses. Recent Prog Horm Res 57, 435–455 (2002). 12017556 10.1210/rp.57.1.435

[b13] Mak I. Y. H. *et al.* Regulated expression of signal transducer and activator of transcription, Stat5, and its enhancement of PRL expression in human endometrial stromal cells *in vitro*. J Clin Endocrinol Metab 87, 2581–2588 (2002). 12050218 10.1210/jcem.87.6.8576

[b14] Yang X. H., Meyer K. & Friedl A. STAT5 and Prolactin Participate in a Positive Autocrine Feedback Loop That Promotes Angiogenesis. J Biol Chem 288, 21184–21196 (2013). 23729680 10.1074/jbc.M113.481119 PMC3774384

[b15] Sultan A. S. *et al.* Stat5 promotes homotypic adhesion and inhibits invasive characteristics of human breast cancer cells. Oncogene 24, 746–760 (2005). 15592524 10.1038/sj.onc.1208203

[b16] Li H. Z. *et al.* Activation of signal transducer and activator of transcription 5 in human prostate cancer is associated with high histological grade. Cancer Res 64, 4774–4782 (2004). 15256446 10.1158/0008-5472.CAN-03-3499

[b17] Muller J., Sperl B., Reindl W., Kiessling A. & Berg T. Discovery of chromone-based inhibitors of the transcription factor STAT5. Chembiochem 9, 723–727 (2008). 18247434 10.1002/cbic.200700701

[b18] Elumalai N., Berg A., Rubner S. & Berg T. Phosphorylation of Capsaicinoid Derivatives Provides Highly Potent and Selective Inhibitors of the Transcription Factor STAT5b. ACS Chem Biol 10, 2884–2890 (2015). 26469307 10.1021/acschembio.5b00817

[b19] Cumaraswamy A. A., Todic A., Resetca D., Minden M. D. & Gunning P. T. Inhibitors of Stat5 protein signalling. Medchemcomm 3, 22–27 (2012).

[b20] Lee H. J., Korshavn K. J., Kochi A., Derrick J. S. & Lim M. H. Cholesterol and metal ions in Alzheimer’s disease. Chem Soc Rev 43, 6672–6682 (2014). 24710074 10.1039/c4cs00005f

[b21] Henke H., Kandioller W., Hanif M., Keppler B. K. & Hartinger C. G. Organometallic Ruthenium and Osmium Compounds of Pyridin-2-and-4-ones as Potential Anticancer Agents. Chem Biodivers 9, 1718–1727 (2012). 22976964 10.1002/cbdv.201200005

[b22] Gasser G. Metal Complexes and Medicine: A Successful Combination. Chimia 69, 442–446 (2015). 10.2533/chimia.2015.442 28482977

[b23] Oehninger L., Rubbiani R. & Ott I. N-Heterocyclic carbene metal complexes in medicinal chemistry. Dalton Trans 42, 3269–3284 (2013). 23223752 10.1039/c2dt32617e

[b24] Leung C. H. *et al.* Metal complexes as inhibitors of transcription factor activity. Coord Chem Rev 257, 3139–3151 (2013).

[b25] Leung C. H., Zhong H. J., Chan D. S. H. & Ma D. L. Bioactive iridium and rhodium complexes as therapeutic agents. Coord Chem Rev 257, 1764–1776 (2013).

[b26] Ma D. L. *et al.* Metal complexes for the detection of disease-related protein biomarkers. Coord Chem Rev 324, 90–105 (2016).

[b27] Leung C. H., Liu L. J., Leung K. H. & Ma D. L. Epigenetic modulation by inorganic metal complexes. Coord Chem Rev 319, 25–34 (2016).

[b28] Liu L. J. *et al.* Inhibition of the p53/hDM2 protein-protein interaction by cyclometallated iridium(III) compounds. Oncotarget 7, 13965–13975 (2016). 26883110 10.18632/oncotarget.7369 PMC4924691

[b29] Lin S. *et al.* Luminescence switch-on detection of protein tyrosine kinase-7 using a G-quadruplex-selective probe. Chem Sci 6, 4284–4290 (2015). 29218197 10.1039/c5sc01320h PMC5707507

[b30] Leung K. H. *et al.* Label-free luminescence switch-on detection of hepatitis C virus NS3 helicase activity using a G-quadruplex-selective probe. Chem Sci 6, 2166–2171 (2015). 28808523 10.1039/c4sc03319a PMC5539802

[b31] Zhong H. J. *et al.* An iridium(III)-based irreversible protein-protein interaction inhibitor of BRD4 as a potent anticancer agent. Chem Sci 6, 5400–5408 (2015). 28757943 10.1039/c5sc02321a PMC5510529

[b32] Liu L. J. *et al.* An Iridium(III) Complex Inhibits JMJD2 Activities and Acts as a Potential Epigenetic Modulator. J Med Chem 58, 6697–6703 (2015). 26225543 10.1021/acs.jmedchem.5b00375

[b33] Frezza M. *et al.* Novel Metals and Metal Complexes as Platforms for Cancer Therapy. Curr Pharm Des 16, 1813–1825 (2010). 20337575 10.2174/138161210791209009 PMC3759287

[b34] Leung C. H., Lin S., Zhong H. J. & Ma D. L. Metal complexes as potential modulators of inflammatory and autoimmune responses. Chem Sci 6, 871–884 (2015). 28660015 10.1039/c4sc03094j PMC5472922

[b35] Pillozzi S. *et al.* NAMI-A is highly cytotoxic toward leukaemia cell lines: evidence of inhibition of KCa 3.1 channels. Dalton Trans 43, 12150–12155 (2014). 24975719 10.1039/c4dt01356e

[b36] Cebrian-Losantos B. *et al.* Osmium NAMI-A analogues: Synthesis, structural and spectroscopic characterization, and antiproliferative properties. Inorg Chem 46, 5023–5033 (2007). 17497853 10.1021/ic700405y

[b37] Maksimoska J. *et al.* Similar biological activities of two isostructural ruthenium and osmium complexes. Chem Eur J 14, 4816–4822 (2008). 18425743 10.1002/chem.200800294 PMC2753370

[b38] Hearn J. M. *et al.* Potent organo-osmium compound shifts metabolism in epithelial ovarian cancer cells. Proc Natl Acad Sci USA 112, E3800–E3805 (2015). 26162681 10.1073/pnas.1500925112 PMC4517206

[b39] Maillet A., Yadav S., Loo Y. L., Sachaphibulkij K. & Pervaiz S. A novel Osmium-based compound targets the mitochondria and triggers ROS-dependent apoptosis in colon carcinoma. Cell Death Dis 4, e653 (2013). 23744353 10.1038/cddis.2013.185 PMC3698552

[b40] Turkson J. *et al.* A novel platinum compound inhibits constitutive Stat3 signaling and induces cell cycle arrest and apoptosis of malignant cells. J Biol Chem 280, 32979–32988 (2005). 16046414 10.1074/jbc.M502694200

[b41] Lai P. S. *et al.* A STAT inhibitor patent review: progress since 2011. Expert Opin Ther Pat 25, 1397–1421 (2015). 26394986 10.1517/13543776.2015.1086749

[b42] Drewry J. A. *et al.* Coordination complex SH2 domain proteomimetics: an alternative approach to disrupting oncogenic protein-protein interactions. Chem Commun 46, 892–894 (2010). 10.1039/b919608k PMC2910512 20107641

[b43] Ma D. L. *et al.* Antagonizing STAT3 Dimerization with a Rhodium(III) Complex. Angew Chem Int Ed 53, 9178–9182 (2014). 10.1002/anie.201404686 24889897

[b44] Tamaki Y., Koike K., Morimoto T., Yamazaki Y. & Ishitani O. Red-Light-Driven Photocatalytic Reduction of CO2 using Os(II)-Re(I) Supramolecular Complexes. Inorg Chem 52, 11902–11909 (2013). 24083376 10.1021/ic4015543

[b45] Jafari R. *et al.* The cellular thermal shift assay for evaluating drug target interactions in cells. Nat Protoc 9, 2100–2122 (2014). 25101824 10.1038/nprot.2014.138

